# Diagnosis of HIV-Associated Oral Lesions in Relation to Early versus Delayed Antiretroviral Therapy: Results from the CIPRA HT001 Trial

**DOI:** 10.1371/journal.pone.0150656

**Published:** 2016-03-01

**Authors:** Ashita S. Batavia, Rode Secours, Patrice Espinosa, Marc Antoine Jean Juste, Patrice Severe, Jean William Pape, Daniel W. Fitzgerald

**Affiliations:** 1 Weill Cornell Medical Center, New York, New York, United States of America; 2 Groupe Haitien d’Etude du Sarcome de Kaposi et des Infections Opportunistes (GHESKIO), Port-au-Prince, Haiti; 3 University of California San Francisco School of Dentistry, San Francisco, California, United States of America; Rega Institute for Medical Research, BELGIUM

## Abstract

Oral mucosal lesions that are associated with HIV infection can play an important role in guiding the decision to initiate antiretroviral therapy (ART). The incidence of these lesions relative to the timing of ART initiation has not been well characterized. A randomized controlled clinical trial was conducted at the GHESKIO Center in Port-au-Prince, Haiti between 2004 and 2009. 816 HIV-infected ART-naïve participants with CD4 T cell counts between 200 and 350 cells/mm^3^ were randomized to either immediate ART initiation (early group; N = 408), or initiation when CD4 T cell count was less than or equal 200 cells/mm^3^ or with the development of an AIDS-defining condition (delayed group; N = 408). Every 3 months, all participants underwent an oral examination. The incidence of oral lesions was 4.10 in the early group and 17.85 in the delayed group (p-value <0.01). In comparison to the early group, there was a significantly higher incidence of candidiasis, hairy leukoplakia, herpes labialis, and recurrent herpes simplex in the delayed group. The incidence of oral warts in delayed group was 0.97 before therapy and 4.27 post-ART initiation (p-value <0.01). In the delayed group the incidence of oral warts post-ART initiation was significantly higher than that seen in the early group (4.27 versus 1.09; p-value <0.01). The incidence of oral warts increased after ART was initiated, and relative to the early group there was a four-fold increase in oral warts if ART was initiated following an AIDS diagnosis. Based upon our findings, candidiasis, hairy leukoplakia, herpes labialis, and recurrent herpes simplex indicate immune suppression and the need to start ART. In contrast, oral warts are a sign of immune reconstitution following ART initiation.

## Introduction

Globally, there are 35.0 million people living with HIV infection. The majority of these individuals live in resource limited settings where initiation of antiretroviral therapy (ART) must occur in a cost conscious manner [1]. Haiti, the poorest country in the Western Hemisphere, also has the highest HIV prevalence in the Caribbean. It is estimated that 2.0% of adults between the ages of 15 and 49 are infected [2]. In this context simple tools such as the oral examination can provide important clinical information about HIV disease progression and the need to initiate ART [3–6].

The appearance of oral lesions in relation to declining CD4 T cell counts, initiation of antiretroviral therapy (ART), and immune reconstitution is complex. Some HIV-associated oral lesions such as candidiasis and hairy leukoplakia are more common as CD4 T cell counts decline and are reversed with immune reconstitution [4,7]. This is in contrast with other oral lesions such as Kaposi sarcoma, which occur more frequently with declining CD4 T cells counts but may not reverse with ART [8,9]. And finally, other lesions such as HPV-associated oral warts may be more common in the context of ART-associated immune reconstitution [6,10,11].

Since HIV-associated oral lesions have diverse etiologies ranging from microbial to malignant to idiopathic [12], it is important to characterize how the timing of ART initiation, and by extension the immune landscape of the host, alters the incidence of these lesions. This information will enhance the utility of the oral examination in an era when our approach to treatment is rapidly changing. The oral examination is also a low cost clinical tool that can guide the initiation of ART. This is particularly important in the context of a flat HIV funding environment and calls for the identification of patients in need of therapy and a rapid expansion in HIV treatment capacity [13,14]

In this study we use randomized clinical trial data is to understand how CD4 T cell counts and the timing of ART initiation affect the incidence of HIV-associated oral lesions.

## Materials and Methods

### Study Setting

The study was conducted at the Center of the Haitian Group for the Study of Kaposi’s Sarcoma and Opportunistic Infections (GHESKIO) in Port au Prince, Haiti. GHESKIO provides no-cost comprehensive HIV care and conducts HIV clinical research.

### Study Design

The Comprehensive International Program for Research on AIDS (CIPRA HT001) was a prospective randomized controlled trial conducted at GHESKIO. The study and consent forms were approved by the institutional review boards at GHESKIO and at the Weill Cornell Medical College [15,16]. 816 participants were enrolled between August 2005 and July 2008. Participants were at least 18 years old with a confirmed CD4 T cell count between 200 and 350 cells/mm^3^, without any history of an AIDS defining illness, and ART-naïve. Participants were randomized to either early ART initiation (CD4 T cell count ≤ 350 cells/mm^3^; N = 408) or delayed ART initiation (CD4 T cell count ≤ 200 cells/mm^3^ or with the development of an AIDS-defining condition; N = 408). Participants in the early ART initiation group were started on treatment within 2 weeks of enrollment. The primary endpoint was survival. In June 2009, a scheduled interim analysis revealed that the trial had crossed the pre-specified stopping boundary for a difference in survival between groups. The data and safety monitoring board recommended that all participants in the delayed ART initiation group be given ART. The 2009 analysis and complete details on study design have been reported [15].

As part of CIPRA HT001 protocol all participants underwent a comprehensive physical examination, including examination of the oral cavity, at enrollment and then every three months for the duration of the study. We report all incident oral exam findings in correlation with the timing of ART initiation. We have included data up to July 31st, 2009.

### Diagnosis of HIV-associated Oral Lesions

Trained research clinicians conducted structured oral examinations. Diagnosis of HIV-associated oral lesions was made using standardized case definitions established by the Oral HIV/AIDS Research Alliance of the AIDS Clinical Trials Group [12,17]. Recurrent aphthous ulceration was diagnosed if the lesions were noted on non-consecutive oral examinations, or if the lesion was noted and the participant reported other similar lesions in the previous 12 months. Recurrent herpes labialis was diagnosed if the herpetic lesions were noted on examinations separated by 6 months, or with documentation of an intervening examination without herpetic lesions. The diagnosis of oral candidiasis includes either erythematous or pseudomembranous candidiasis. Only incident diagnoses made after enrollment and on or before 7/31/2009 were considered for analysis.

### Statistical Analysis

Clinical and laboratory information were entered electronically in Haiti and managed by Frontier Science and Technology Research Foundation in Amherst, New York. Data was exported into Microsoft Excel for cleaning and analysis was performed using Stata 13 software (StataCorp LP, TX). All analyses were based on intention to treat.

Primary data analysis was conducted from the time of randomization, and secondary analysis in the delayed ART initiation group was conducted from the time of ART initiation. The probability of survival without development of an incident HIV-associated oral lesion was calculated using standard Kaplan-Meier survival methods and differences between the curves were evaluated using the Hazard Ratios. Incidence rates were calculated as the number of new cases per 100 person-years of follow-up. For comparison of rates, we used Fisher’s exact test. Two-sided hypotheses and tests were adopted for all statistical inferences.

We determined which HIV-associated oral lesions occurred more frequently in the early versus delayed ART initiation groups. Among participants in the delayed group, HIV-associated oral lesions were further stratified by their occurrence in the pre- vs. post-ART period. We also report the median CD4 T cell count with interquartile range (IQR) in cells/mm^3^ at the time that the oral lesion was diagnosed.

## Results

### Study Population

The 816 participants in the CIPRA HT001 trial underwent 1:1 randomization to the early and delayed ART initiation groups. The enrollment characteristics of participants in each group were similar [15]. At enrollment median age was 40 years, 58% were female, median BMI was 21.2 kg/m^2^, median CD4 T cell count was 281 cells/mm^3^, and 32.0% of all participants were asymptomatic, or World Health Organization (WHO) HIV Clinical Stage 1 [18]. Eligibility was limited to subjects with WHO HIV Clinical Stage 1, 2, or 3 only.

### Status at the Time of Analysis

All 408 participants randomized to the early group started therapy within 2 weeks of enrollment; there were a total of 9 deaths and 23 were lost during the study period. In the early group the median follow up time was 2.0 years and the total follow up time was 828.9 years. Among the 408 participants in the delayed group, 27 died and 19 were lost before ART, and an additional 7 died and 7 were lost after ART was initiated. Only 244 (59.8%) participants in the delayed group started therapy during the study period and their median time from enrollment to ART initiation was 1.2 years. In the delayed group the median follow up time was 2.0 years and the total follow up time for was 829.0 years. In the delayed group the total follow up time prior to therapy was 618.3 years, and the total follow up time after ART initiation was 210.7 years.

### Outcomes

There were 182 incident HIV-associated oral lesions diagnosed in 163 participants during the study period. Among the 408 participants in the early group, 34 oral lesions were diagnosed. By contrast, among the 408 participants in the delayed group, 148 oral lesions were diagnosed. By Kaplan-Meier estimates, participants in the delayed group were more likely to be diagnosed with an HIV-associated oral lesion (HR 4.19, 95% CI: 2.78–6.34; p-value <0.01).

### Enrollment Predictors

At enrollment, participants with incident oral lesions did not differ in age (median 40 years), gender (56% female), median BMI (20.0 kg/m^2^), or CD4 T cell count (275 cells/mm^3^; p-value 0.62). At enrollment, participants with advanced HIV (WHO Clinical Stage 2 or 3) were more likely to develop an oral lesion than participants with early HIV (WHO Clinical Stage 1; HR 1.61, 95% CI: 1.07–2.41; p-value 0.02).

### Incidence of HIV-associated Oral Lesions

Table 1 reports incidence per 100 person-years. The incidence of oral lesions was 4.10 in the early group and 17.85 in the delayed group (p-value <0.01). In comparison to the early group, there was a significantly higher incidence of candidiasis (p-value <0.01), leukoplakia (p-value 0.02), and herpes labialis (p-value 0.05) in the delayed group. In the delayed group the incidence of oral lesions prior to ART initiation was 21.51, and the incidence after therapy was 7.11 (p-value <0.01).

**Table 1:** Incident HIV-associated oral lesions in the early and delayed ART initiation groups.

	Early ART initiation group (N = 408)^a^		Delayed ART initiation group (N = 408)^b^		p-value	Delayed group, pre-ART initiation (N = 408)^c^		Delayed group, post-ART initiation (N = 245)^c^		p-value
	N	Incidence	N	Incidence		N	Incidence	N	Incidence	
**Fungal Infections**										
Candidiasis	14	1.69	89	10.74	<0.01	86	13.91	3	1.42	<0.01
Angular chelitis	2	0.24	7	0.84	0.18	7	1.13	0	0.00	0.20
**Viral Infections**										
Hairy leukoplakia	1	0.12	9	1.09	0.02	9	1.46	0	0.00	0.12
Oral warts	9	1.09	15	1.81	0.21	6	0.97	9	4.27	<0.01
Herpes labialis	6	0.72	15	1.81	0.05	13	2.10	2	0.95	0.38
Recurrent intraoral herpes simplex	0	0.00	6	0.72	0.03	6	0.97	0	0.00	0.35
**Idiopathic**										
Recurrent aphthous stomatitis	1	0.12	3	0.36	0.32	3	0.49	0	0.00	0.57
**Neoplasms**										
Oral Kaposi sarcoma	1	0.12	4	0.48	0.37	3	0.49	1	0.47	0.98
**Total**	34	4.10	148	17.85	<0.01	133	21.51	15	7.11	<0.01

^a^ There were 408 participants randomized to the early ART initiation group; the total follow up time for the early group was 828.86 years. Incidence is reported as new diagnoses per 100 person-years.

^b^ There were 408 participants randomized to the delayed ART initiation group; the total follow up time for the delayed group was 828.96 years. Incidence is reported as new diagnoses per 100 person-years.

^c^ In the delayed ART initiation group, 244 out of 408 participants started ART before July 31^st^, 2009. In the delayed group, the total pre-ART initiation follow up time was 618.25 years and the total post-ART follow up time was 210.71 years. Incidence is reported as new diagnoses per 100 person-years.

In the delayed group, the incidence of candidiasis was significantly higher before therapy was started (13.59 versus 1.42; p-value <0.01), and occurred at a median CD4 T cell count of 259 cells/mm^3^ (IQR 222–309 cells/mm^3^). In the post-ART initiation period the incidence of candidiasis was similar to that seen in the early group (1.42 versus 1.69). In the post-ART period the median CD4 T cell count at the time of a candida diagnosis was 368 cells/mm^3^ (IQR 222–309 cells/mm^3^), and the median time from ART initiation to a diagnosis of candidiasis was 554 days (IQR 172–753 days).

The incidence of oral warts in delayed group was 0.97 before therapy (median proximate CD4 T cell count 261 cells/mm^3^, IQR 232–314 cells/mm^3^) and 4.27 post-ART initiation (median proximate CD4 count 375 cells/mm^3^, IQR: 290–455 cells/mm^3^; p-value <0.01). In the delayed group the incidence of oral warts post-ART initiation was significantly higher than that seen in the early group (4.27 versus 1.09; p-value <0.01). The median time from ART initiation to a diagnosis of oral warts was 375 days (IQR 152–573 days).

## Discussion

Among participants in the CIPRA HT001 trial, careful examination of the oral cavity provided valuable information on HIV disease progression and falling CD4 T cell counts that guided ART initiation in 20.0% of the cohort. Based upon our findings, candidiasis, hairy leukoplakia, herpes labialis, and recurrent herpes simplex indicate immune suppression and the need to start ART. In contrast, oral warts are a sign of immune reconstitution after ART initiation. The incidence of oral warts increased after ART was initiated, and relative to the early group there was a four-fold increase in oral warts if ART was initiated at a CD4 T cell count less than or equal to 200 cells/mm^3^ or following diagnosis of an AIDS-defining condition.

There were 182 incident HIV-associated oral lesions during the study period. In the delayed group there was a significantly higher incidence of oral candidiasis, hairy leukoplakia, herpes labialis, and recurrent herpes labialis. In the delayed group, stratification of these oral lesions by occurrence before or after ART initiation showed that after therapy the incidence approached that seen in the early group. Diagnosis of oral candidiasis or hairy leukoplakia after starting ART may be indicative of viral replication [3,4,19,20] and warrants careful evaluation of medication adherence and perhaps the emergence of resistance.

Multiple studies have shown an increase in HPV-associated oral lesions with the advent of ART and have speculated that oral warts are part of the immune reconstitution inflammatory syndrome (IRIS) [5,10,21,22]. Advanced immunosuppression, the criteria for starting therapy in the delayed group, is a known risk factor for IRIS and may account for the increased incidence of oral warts in the delayed group [23]. In the delayed group, waiting until a participant’s CD4 T cell counts fell below 200 cells/mm^3^ or they development of an AIDS-defining condition may have increased the likelihood that a critical irreversible threshold for developing HPV-related disease was exceeded [24]. In the delayed group the median time from randomization to ART initiation was 1.15 years. This additional exposure to unchecked HIV viral replication, possible HPV acquisition, and HPV/HIV interaction from HIV *tat* protein could have increased the risk of HPV proliferation in the delayed group [25,26]. Oral warts in the post-ART period occurred at a median CD4 T cell count of 375 cells/mm^3^ and were indicative of an immunologic response to therapy.

The oral examination is a low cost tool that can be used to HIV disease progression and response to therapy. Oral candidiasis and hairy leukoplakia are indicative of viral replication and immunosuppression, whereas oral warts suggest immune reconstitution. Advanced immunosuppression with CD4 T cell count ≤200 cells/mm^3^ or development of an AIDS defining condition prior to starting ART results in a four-fold higher incidence of oral warts as the immune system recovers.

## Supporting Information

S1 Data Data Set for HIV-associated Oral lesions from the CIPRA HT001 Trial. (XLSX)
